# Supplementary material for: Capturing chemical intuition in synthesis of metal-organic frameworks
Source: Nat Commun. 2019 Feb 1;10:539. doi: 10.1038/s41467-019-08483-9 (PMC6358622; doi:10.1038/s41467-019-08483-9)
Supplement: Supplementary file 1 — Supplementary Information [file 41467_2019_8483_MOESM1_ESM.pdf]

Supporting Information

Capturing chemical intuition in synthesis of metal-organic frameworks

Moosavi et al.

Supplementary Note 1. Genetic algorithm

Genetic algorithms (GA) are global search methods and the aim of a GA is to search the phase space constructed by the optimisation variables to find the global optimum of the objective function (1). In a GA optimisation, the value of the objective function is evaluated by a population of individual explorers (chromosomes) distributed in the phase space. Each individual explorer is uniquely defined by its genes, which are the values of the optimisation variables. After evaluation of the objective function, GA randomly selects the good performing individuals to reproduce and create children in the form of a new generation of explorers by the crossover operation that combines parents to generate new children. Furthermore, to explore the not seen parts of the phase space, mutation happens. Hence, a new generation proposed by a GA is a combination of samples with good genes, with a controlled number of new genes. The quality of genes of explorers evolve toward an optimal solution over successive generations.

Here, we have implemented an adaptive genetic algorithm for synthesis of MOFs (Supplementary Figure 1 blue block). All the codes are adapted from MATLAB global optimisation toolbox (2). The GA probes the constrained chemical phase space constructed by the nine synthesis variables listed in Supplementary Table 1. For the synthesis of HKUST-1 we have ensured that our range includes the successful synthesis conditions that are reported in the literature. The volume of solvent was allowed to vary between 1 to 6 ml, as an implicit function of the solvent composition, where 6ml is the maximum volume allowed by the robot. The population size of the GA was fixed to 30 chromosomes for each generation.

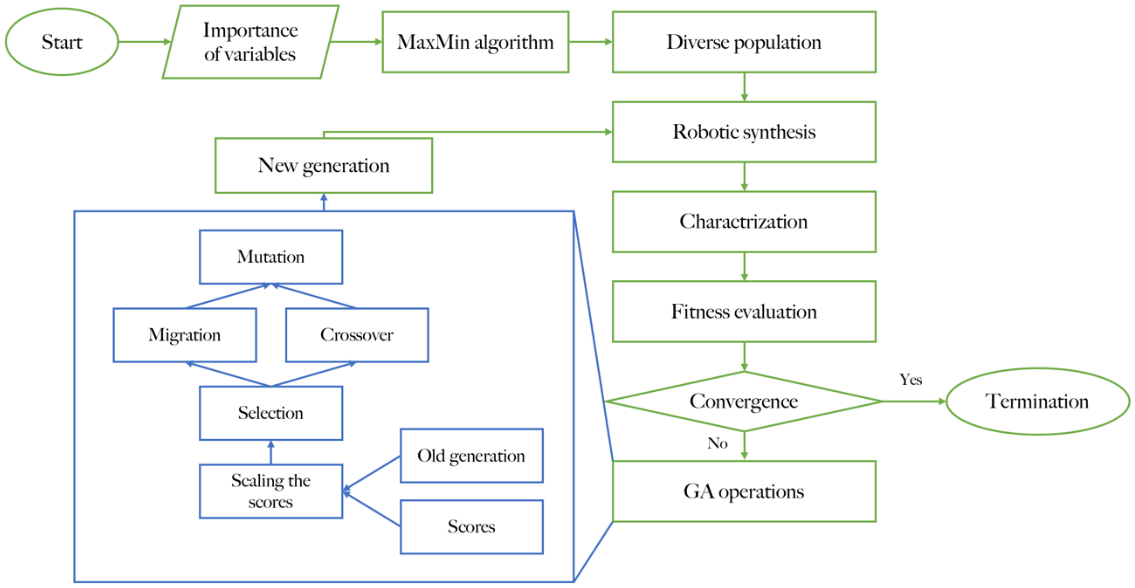

Supplementary Figure 1. Flowchart representation of the procedure used for synthesis of MOFs.

To generate a new generation, the GA takes the chromosomes of the past generation and their corresponding fitness score based on an objective function. The fitness functions in current study are crystallinity or crystallinity and BET surface area of the first and the second generations, respectively. We use the full width at half maximum of powder X-ray diffraction patters as a measure of crystallinity of samples (see below). The algorithm scales the scores with their ranking using $1/\sqrt{r}$, where r is the rank of each chromosome using tied rank for similar performing chromosomes. After scaling the scores, the GA produces 30 new children using the migration and crossover, constituting 10 percent and 90 percent of population, respectively. In migration operation, the genes of the top performing samples from old generation are transformed to the new generation. We use intermediate crossover function to respect the linear constrained of the optimisation. In the intermediate crossover scheme, the child is created using the weighted average of the parents, i.e. $\text{child} = \text{parent1} + \text{random number} * (\text{parent1} - \text{parent2})$. The chance of being selected as a parent by the algorithm for crossover operation is proportional to the scaled score, i.e. the rank of chromosomes. The final step in the GA is to mutate the genes of the new chromosomes. This

step is crucial to explore the not-seen part of the chemical space. We use gaussian mutation function where the mutated genes are chosen within a Gaussian distribution around the unmutated gene with a shrinking standard deviation of

$$\sigma = S\sigma_0, \quad (1)$$

where $S = \frac{1}{Generation\ number}$ is the shrink factor and the initial standard deviation σ_0 was set to 0.2 of ranges of variables. An illustrative example of the genetic operation is shown in Supplementary Figure 2.

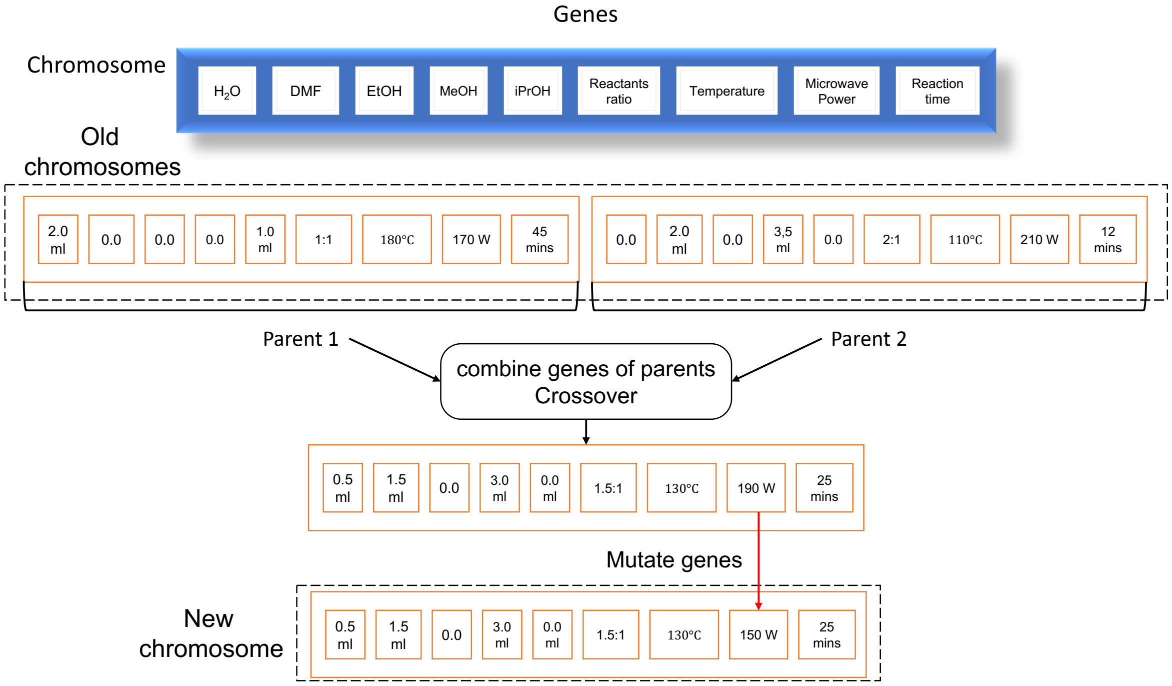

Supplementary Figure 2. A representative experimental condition and its transformation to the consecutive generation. Each experimental variable is a gene in a chromosome which is an experimental trial. The genetic algorithm operations generate new children using the genes of parents.

The optimisation is initialized with the set of most diverse synthesis conditions based on the MaxMin algorithm (See section Supplementary Note 2. MaxMin procedure). Starting with the set of most diverse genes and keeping a decent mutation rate are essential for efficient exploration of the chemical space and finding global optimum of the objective function.

The crystallinity of each sample was assessed by the full width at half maximum (FWHM) of powder X-ray diffraction patterns (PXRD) (3–5). We start with separating peaks in the PXRD. Afterwards, a Gaussian function is fitted to the peak which give us the FWHM with the following set of equations:

$$f(x) = \frac{1}{\sigma\sqrt{2\pi}} \exp\left[-\left(\frac{(x-x_0)^2}{2\sigma^2}\right)\right], \quad (2)$$

$$\mathrm{FWHM} = 2\sqrt{2\ln(2)}\sigma, \quad (3)$$

where variable x is the 2θ of the diffraction angel. The average FWHM of all the peaks of the PXRD is taken as the measure of crystallinity. Lorentzian, Pearson, and combined Lorentzian, Pearson and Gaussian distributions were also considered, and no considerable differences were observed in the ranking.

For the selection step, GA only takes the ranking of the performance of individuals in the current population, and therefore, its objective function can easily be adapted for more optimization's goals, e.g. reaction yield, crystal morphology, etc. Moreover, adding or removing synthesis variables and conditions is straightforward.

**Synthesis variable**	Optimisation constraints	Notes
**Water (H_2O)**	0 − 6 ml	The total solvent volume is constrained to be between 1ml to 6 ml.
**Dimethylformamide (DMF)**	0 − 6 ml	
**Ethanol (EtOH)**	0 − 6 ml	
**Methanol (MeOH)**	0 − 6 ml	
**Isopropyl alcohol (iPrOH)**	0 − 6 ml	
**Reactants ratio**	0.8 − 1.8	Molar ratio of Cu nitride to BTC ligands
**Temperature**	100 − 200 °C	
**Microwave power**	150 − 250 W	
**Reaction time**	2 − 60 mins	

Supplementary Table 1. The synthesis variables constructing the chemical phase space, and their corresponding optimisation range.

Supplementary Note 2. MaxMin procedure

To initiate the optimisation cycle, one needs to an initial guess for the synthesis variables. Since we do not know the landscape of the chemical space a priori, the best way of initialization is to have a homogenous coverage of the entire chemical space. Particularly for the GA, we would like to ensure that the initial population includes the most diverse combination of genes. Indeed, since we have few samples in the initial population, methods like random initialisation or human guided guessing can introduce a high bias in the initial population. Thus, we have use the MaxMin procedure which ensures having the most diverse set of initial synthetic conditions (6). The first landmark point is chosen at random in the phase space. The subsequent landmark is the data point in the phase space with the maximum dissimilarity to its most similar landmark point form all the previous landmarks. In other words, the data point that has the maximum distance to its closest landmark will be added to the landmarks set. This procedure continues till all the landmarks are chosen, here the size of the landmarks set is 30.

This procedure relies on assigning pairwise distance between data points. We use Euclidean distance metric based on the normalized value of variables to the range of their feasible changes (look Supplementary Table 1 for ranges). In the Euclidean distance calculations, we can easily incorporate the weighted importance of variables. The following equation was used to calculate the pairwise distances between two data points, a and b:

$$d_{a,b} = \sqrt{\sum_i^n w_i (a_i - b_i)^2}, \quad (4)$$

where n is the total number of variables and w_i is the relative importance of variable i.

Supplementary Note 3. Machine learning

In this work, we use machine learning for two purposes, namely capturing chemical intuition and prediction of outcome of synthesis. Due to the modular architecture of the developed approach for MOF synthesis, incorporation of other abilities to the current framework is straightforward. In the following, we first explain the methodology used for machine learning. Afterwards, we explain how this trained machine can be used for each of the two purposes.

The core machine learning method in this work is random decision forest (RF) (7,8). We train a random decision forest regression model to predict the outcome of MOF synthesis based on the synthesis variables as inputs. All the machine learning codes were adapted from MATLAB (2). Crystallinity and phase purity were used as the measure of outcome of experiment (See Supplementary Note 1. Genetic algorithm for details). We use bootstrapped-aggregate random forest to decrease variance and overfitting of the model, where the predictors and training data set are chosen randomly with replacement (in and out of bag training data) and the prediction of the model is based on the average prediction of all the decision trees in the forest. The accuracy of the regression model was assessed by comparing the predictions of the model with actual outcome of experiments using cross-validation and not seen data points. In cross-validation, the training set is split to two complementary subsets, one with large population (90% of the entire set) and a small one with the remaining data in the training data set (10% of the entire set). The model is trained with the large subset and the small subset is used for validation. This procedure was repeated several times and the accuracy of the model is assessed by averaging the mean absolute error (MAE) in the predictions. To analyse the sensitivity of the accuracy to the size of the training set, we performed 40 extra experiments (Supplementary Figure 3).

Other machine learning methods including support vector machine (SVM) and artificial neural networks (ANN) were also used for comparison. Both methods give comparable

accuracy to RF, and therefore, we use RF for the rest of this study due to simplicity in interpretation of results, particularly the relative importance of variables.

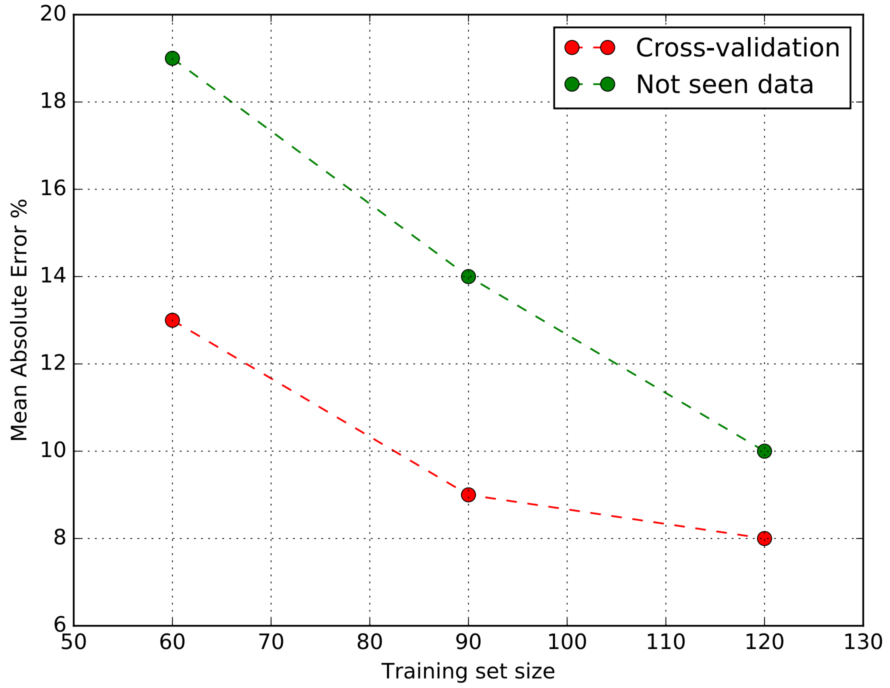

Supplementary Figure 3. The learning curve for random decision forest regression model. Increasing the size of training set decreases the error in predictions of crystallinity of samples. The data are from the experiments on HKUST-1

Supplementary Note 4. *In silico* prediction of synthesis outcome

The trained model can be used for prediction of the outcome of synthesis without performing the experiments. For data we collected for H-KUST1, the mean-absolute error (MAE) of the trained model for training size of 90 is 9% and 14% for cross-validation and new data points, respectively, which are indeed sufficient and satisfactory for estimating the outcome of a synthesis. Particularly, this prediction is useful to eliminate many chemical hypothesis (synthesis conditions to be tried) that would not yield any favourable outcome.

Moreover, this predictive model can be used to boost the convergence of the GA optimisation for synthesis of difficult MOFs. Since GA takes only the last generation into consideration to propose new synthesis conditions, by construction, after some generations, the algorithm might visit the already seen regions of space. This known weakness is associated to the deficient learning of the GA from the previous failed experiments.

Here, we propose a combined GA with machine learning to address this weakness. After each generation, we train the machine learning model, e.g. RF, and monitor the MAE of the prediction. We can rely on the prediction of the machine learning model after some generations when the MAE decreases to a satisfactory rate. Afterwards, we perform an *in silico* evaluation of the outcome of chemical hypothesis proposed by the GA for several generations until they converge to a set of optimal synthesis conditions based on the machine learning model predictions. Then, we experimentally synthesize the *in silico* predicted optimal conditions. The new experimental data is used to further refine the regression model, and the process is repeated until the objective function is satisfied (*Supplementary Figure 4*). This procedure can save many experiments in the intermediate steps and help the GA to converge faster. Indeed, this procedure only required if one needs many GA steps to find satisfactory synthesis conditions.

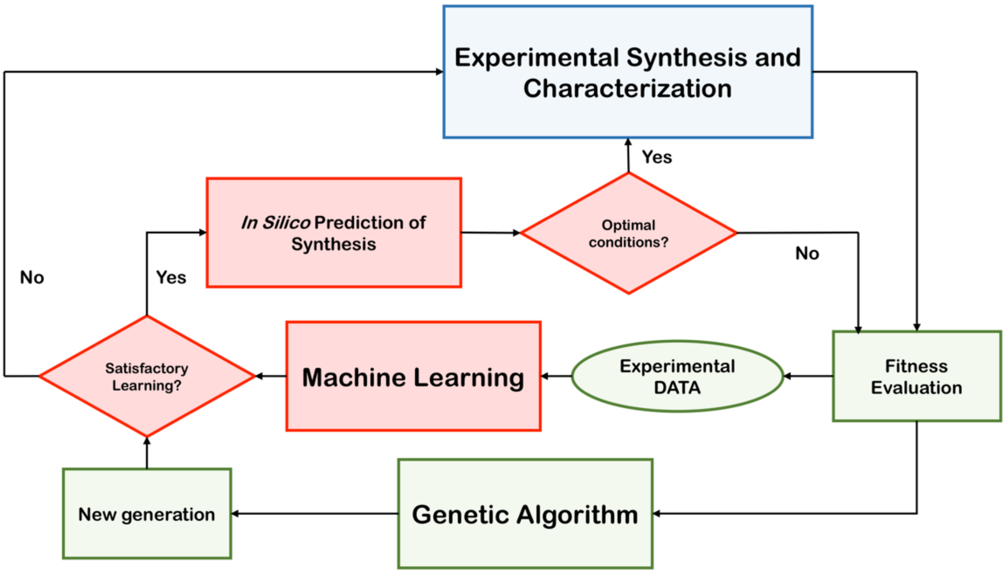

Supplementary Figure 4. Flowchart of the MOF synthesis accelerated by machine learning.

Supplementary Note 5. Capturing the chemical intuition

The essence of chemical intuition is how experts in MOF synthesis weight different synthesis variables while exploring chemical phase space. Indeed, an expert does not blindly modify the synthesis variables, but he or she takes advantage of the accumulated experience on the importance of the different synthesis variables on crystallization and crystal growth in MOF chemistry. Here, we use the trained RF model to estimate the importance of variables based on the difference in the out-of-bag error by scrambling each variable in the out-of-bag observations, i.e., randomly permuting the variable across the out-of-bag data points. The difference is large for important variables, while, permuting a meaningless variable does not lead to a high difference in the out-of-bag error.

Supplementary Note 6. The Sy-Co-Finder web application

We gathered the single programs we developed for this study in SyCoFinder, a web application for finding and optimizing synthesis conditions for MOFs. The application is available on Materials Cloud (9). The application has three main components, compute diverse set, optimize synthesis conditions using genetic algorithm, and estimating importance of variables using machine learning. The genetic algorithm and machine learning codes were ported to python for the web application, using DEAP (10) and scikit-learn (11) packages, respectively. In the supplementary movie 1, we show how the application can be used for synthesis of new MOFs following these steps:

1. Define the chemical space to be explored
2. Generate a diverse set of synthesis conditions with/without chemical intuition (using variable importance)
3. Perform experiments and record fitness of samples
4. Optimize synthesis conditions using genetic algorithm
5. Learn importance of variables using random forest

Supplementary Note 7. On efficiency of sampling in the weighted space

To estimate the gain in efficiency of sampling the chemical space when one takes the importance of variables into account, it is instructive to look at the probability of finding a successful region in the space. For the N experiments homogenously distributed in the space using the MaxMin procedure, the probability of finding such a region in the space is proportional to N/V_{US} where V_{US} is the volume of the unweighted space. Using the importance of variables, each dimension of the chemical space resizes, i.e., the space shrinks on the dimensions with low importance. If one devise the same experiment in the weighted space, the chance of success is now proportional to N/V_{WS}, with $\mathrm{V_{WS}}$ being the volume of weighted space. We know that the gain in sampling efficiency is the ratio of the volumes, i.e. $G = \frac{V_{\mathrm{US}}}{V_{\mathrm{WS}}} = \prod_1^n 1/\, w_{\mathrm{i}}$.

For the chemical space in our study, the gain in success is G ~ 20’000 using the importance of variables from the machine learning. In other words, for the same number of experiments, one has enormously higher chance of finding a successful condition in the weighted space than when there is no intuition developed. Of course, this number is purely mathematical as we assumed a continuous space with infinite resolution in sampling, while for chemical synthesis, a discrete space with finite resolution is more realistic. However, both cases are equivalent as one would save many trials by not changing the unimportant variable during the exploration of the chemical space.

Supplementary Note 8. Importance of variables: other techniques

Feature selection techniques can be classified broadly into three categories: filter methods, wrapper methods and embedded methods (12,13). Univariate filters use a scoring function to rank the importance of each variable based on its correlation with the objective function. Due to their simplicity and low cost, filter methods are widely used. However, considering each variable individually ignores relationships between variables.

Wrapper and embedded methods instead consider the predictive power of subsets of variables taken together, allowing them, for example, to detect redundancy arising from correlation between variables. In wrapper methods, the trained machine learning model is treated as a black box to assess the usefulness of subsets of variables in the prediction performance of the model. Embedded methods perform similar analysis; however, it is performed during the training. For small datasets, embedded methods are recommended over the wrapper methods as they do not require splitting the data to training and validation sets, and consequently, they use the data more efficiently (12). Therefore, random forest embedded method was chosen for this work, which is compared to filter methods below.

F-test and mutual information are two widely used scoring functions in univariate filter methods (12). Supplementary Figure 5 shows how these tests weight the importance of variables. Both filters rank H_2O as the most important variable, in agreement with random forest, while iPrOH is recognized by random forest and F-test but not by mutual information. In Supplementary Figure 6 we plot the crystallinity as a function of synthesis variables to see how the crystallinity is correlated to each variable.

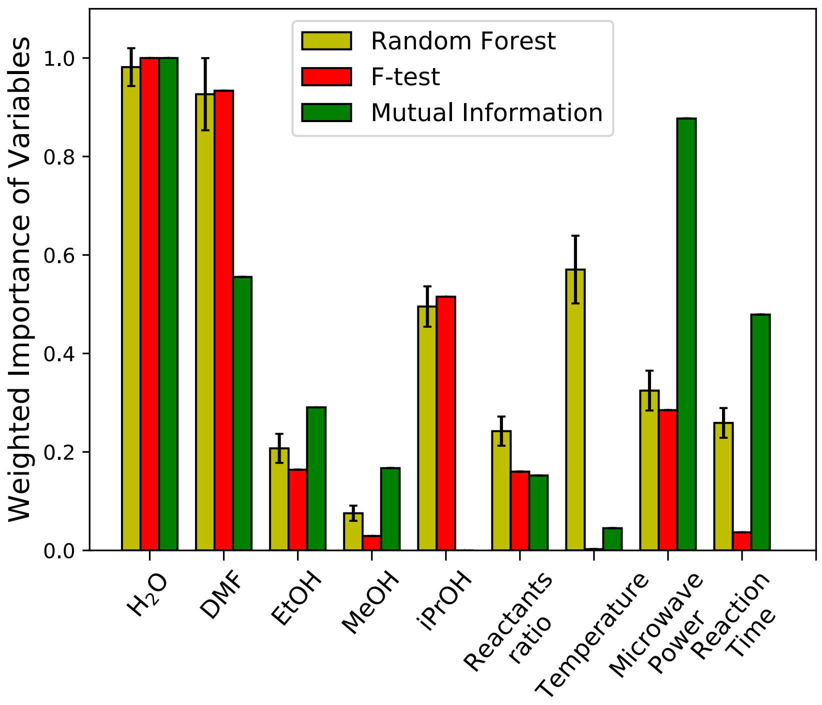

Supplementary Figure 5. The weighted importance of variables computed with univariate filtering.

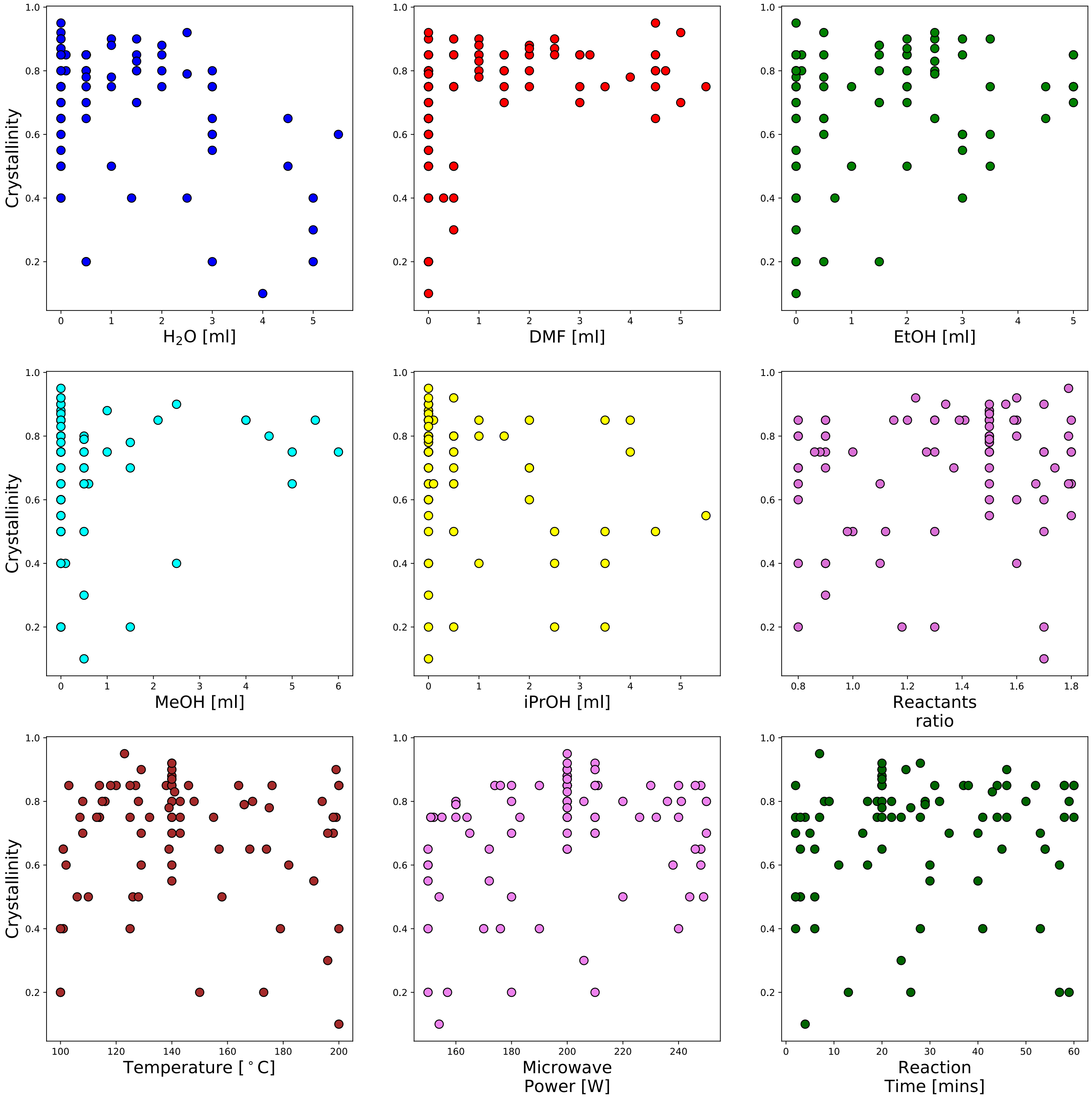

Supplementary Figure 6. The crystallinity of the Cu-HKUST-1 samples as a function of the 9 synthesis variables.

Supplementary Note 9. The timeline of HKUST-1 synthesis

Several synthetic routes for HKUST-1 synthesis have been established and reported. Room temperature (RT) synthesis, conventional electric heating (CEH), microwave (MW), electrochemistry (EC), mechanochemistry (MC), and ultrasonication (US) methods are the commonly employed methods for the synthesis of HKUST-1. The prime objective is to identify the optimum synthetic conditions, which would enable the isolation of crystals or a micro crystalline (long range order), phase pure and porous HKUST-1 framework. Using these different synthesis methods, crystalline phase pure HKUST-1 could be obtained, however, despite the same activation procedure, they show a wide range of BET surface areas (*Supplementary Figure 7*).

From Supplementary Table 2, it can be observed that majority of the research groups have used CEH heating for the synthesis of HKUST-1. Ian D. Williams et. al from Hong Kong University of Science and Technology (17) reported their synthetic conditions which was later modified by several other research groups. 1.8 mM of cupric nitrate trihydrate was heated with 1.0 mM of benzene-1,3,5-tricarboxylic acid (BTC) ligand in 12 ml of 50:50 H_2O: EtOH (EtOH, ethyl alcohol) at 180°C for 12 hours in a Teflon-lined 23-ml Parr pressure vessel which gave turquoise crystals up to dimensions of 80 μm.

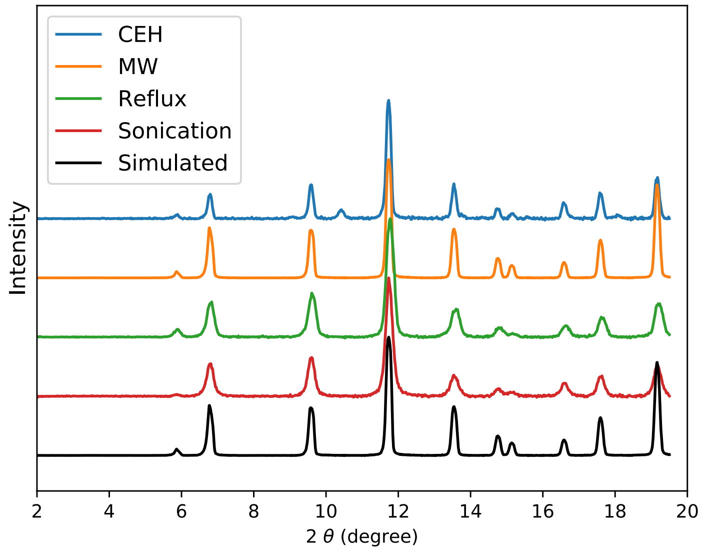

Supplementary Figure 7. The powder X-ray diffraction of the crystalline HKUST-1 synthesized using different methods. These samples have a wide range of BET surface areas: 389 m^2 g^{-1} for CEH; 1228 m^2 g^{-1} for MW, 900 m^2 g^{-1} for reflux, 1036 m^2 g^{-1} for sonication.

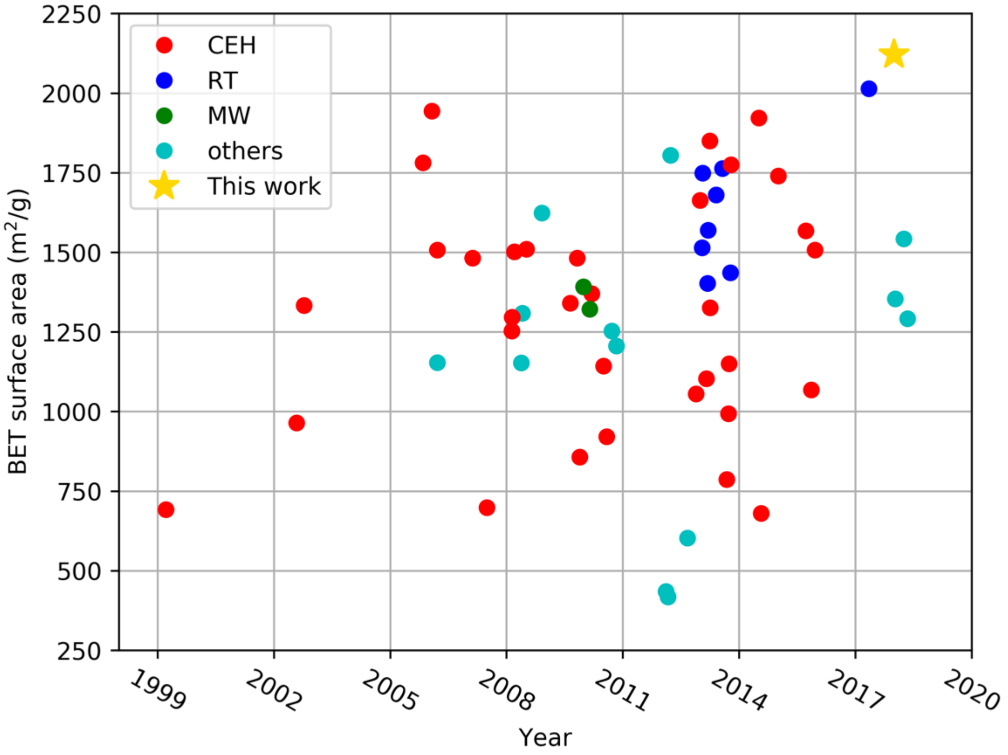

Supplementary Figure 8. Considerable amount of data lost in the literature as only successful synthesis conditions were reported for these data points (~50 data points). The reported BET surface area by many synthesis groups shows a wide range of variations. The horizontal axis (year) of the data points slightly shifted to avoid overlap.

Corresponding Author	BET (m^2g^{-1})	Synthesis technique	Temp (°C)	Time (h)	Solvents	Metal, Ligand	Ref
**Ian D. Williams**	692	CEH	180	12	H_2O, EtOH	1.8 mM<br>1.0 Mm	(17)
**Bein T**	-	High-throughput CEH	180	16	H_2O, EtOH	0.349 g<br>0.1785 g	(18)
**M. Douglas LeVan**	921	CEH	85	24	DMF, H_2O, EtOH	10 g, 5 g	(19)
**R. Staudt**	1270 classical range<br>1555 new	commercially available Basolite™ C300	-n/a-	-n/a-	-n/a-	-n/a-	(20)
**Stefan Kaskel**	1239	Reflux					(21)
**Stefan Kaskel**	1370	CEH	100	24	DMF, H_2O, EtOH	3.5 g, 2.1 g	(22)
**Johan A. Martens**	999 – freeze dried<br><br>1805 – pre cooled	Freeze drying,<br><br>pre cooling,<br><br>RT synthesis<br><br>CEH	-196,<br><br>-60<br><br>25<br><br>180	-n/a-<br><br>-n/a-<br><br>24<br><br>16	EtOH<br><br><br><br><br><br>H_2O, EtOH	1.2 g, 0.6 g<br><br>1.724, 0.5 g<br><br>1.724 g, 0.6 g<br><br>0.349 g, 0.1785 g	(23)
**Hua Kuo**	1055	CEH	110	18	DMF, H_2O, EtOH	6g, 3g	(24)
**Sasidhar Gumma**	1663	CEH	100	10	DMF, H_2O, EtOH	2.077g, 1.0 g	(25)
**J. Karl Johnson**	1482 – MeOH treated<br>698<br><br>Methylene chloride treated	CEH	85<br><br><br><br>85	10 + 8 stirring<br><br>20	DMF, EtOH	2.0 g, 1.0 g<br><br><br><br>2.0 g, 1.0 g	(26)
**Omar M. Yaghi**	1507	CEH	85	24	DMF, H_2O, EtOH	10 g, 5 g	(27)
**Philip L. Llewellyn**	1850	CEH	No info	No info	No info	No info	(28)
**Stefan Kaskel**	1502	CEH	120	24	H_2O, EtOH	3.5 g, 2.1 g	(29)
**Stefan Kaskel**	1340	CEH	100	20	H_2O, EtOH	3.5 g, 2.1 g	(30)
**Florian O. R. L. Mertens**	418 for synthesis at 298 K	non-CEH					(31)

	435 synthesis at 333 K<br><br>603 synthesis at 373 K						
**Zhong Li**	1568.5	CEH	110	24	H_2O, EtOH	0.716 g, 0.421 g	(32)
**Darren Bradshaw**	$Cu(NO_3)_2$ 1403<br><br>$Cu(OAc)_2$ 1436<br><br>$Cu(OAc)_2$ 1514<br><br>$Cu(OAc)_2$ 1763<br><br>$Cu(OAc)_2$ 1560<br><br>$Cu(OH)_2$ 1749<br><br>Basolite C300 1680	RT synthesis					(33)
**Ling-Guang Qiu**	1326	CEH	120	12	H_2O, EtOH	1.093 g, 0.525 g	(34)
**Ralph T. Yang**	1150	CEH	85	20	DMF, H_2O, EtOH	1.0 g, 0.5 g	(35)
**Omar M. Yaghi**	1944	CEH	85	24	DMF, H_2O, EtOH	10 g, 5 g	(36)
**Zifeng Yan**	1922	CEH	75	24	DMF	5 g, 2.5 g	(37)
**Ana M. Afonso**	1068	CEH	110	24	H_2O, EtOH	1.255 g, 0.630 g	(38)
**R. Chirone**	680	CEH	85	21	DMF, H_2O, EtOH	20 g, 10 g	(39)
**Xiaolei Fan**	1507	CEH	100	No info	H_2O, EtOH	0.875 g, 0.42 g	(40)
**Jessica Semanscin**	964.5 large scale synthesis<br><br>1333 small scale synthesis	CEH	150	18	H_2O, EtOH	108.6 g, 49.1 g	(41)
**Omar M. Yaghi**	1781	CEH	85	24	DMF, H_2O, EtOH	10 g, 5 g	(42)
**Ulrich Müller**	1154	EC					(43)
**Alex Wagener**	1510	CEH,	120	14	EtOH	1.75 g, 0.84 g	(44)

	1253<br>1624<br>1309<br>1153	reflux,<br>EC	120	14	H_2O, EtOH	1.75 g, 0.84 g	
**Michael Mehring**	1143<br>1321<br><br>1206<br>1499<br><br>1253<br>424<br><br>1119<br>1421<br><br>897<br>1270	CEH<br>MW<br><br>US<br><br><br>MC<br><br><br>EC<br><br><br>Reflux	120<br>180<br><br>25<br><br><br>25<br><br><br>40	24<br>0.3<br><br>1<br>pulse<br>mode<br>50%<br>power<br>95%<br><br>0.2<br><br><br>30 Hz	H_2O, EtOH<br>H_2O, EtOH<br><br>H_2O, EtOH<br><br><br>No solvent	984 mg, 571 mg<br><br>0.22 g, 0.128 g<br><br>200 mg, 140 mg<br><br>1000 mg, 702 mg	(45)
**Jong-San Chang**	1392	MW	140	1, 300 W	H_2O, EtOH	3.65 mmol, 2 mmol	(46)
**Ralph T. Yang**	1296	CEH	85	20	DMF, H_2O, EtOH	10 g, 5 g	(47)
**Xia Jiang**	1100	US					(48)
**Alexandra Navrotsky**	1775.6	CEH	85	20	DMF, H_2O, EtOH	3:2 molar ratio	(49)
**Nak Cheon Jeong**	1740<br>1690	CEH	80	20	DMF, H_2O, EtOH	0.87 g, 0.22 g	(50)
**Chenguang Liu**	1103.7<br>787<br>993.6	CEH	120	24	H_2O, EtOH	1.5 mmol, 1.0 mmol	(51)
**Praveen K. Thallapally**	2014	RT synthesis	25	24	MeOH	20 mmol, 10 mmol	(52)
**Sasidhar Gumma**	857 for 140°C synthesis<br><br>1482 for 100°C synthesis	CEH	140<br><br>100	48 +16 of stirring<br><br>10 h	DMF, H_2O, EtOH	4.48 g, 1.9664 g<br><br>2.077 g, 1.0 g	(53)
**Tao Wu**	1292.3<br>1354.6<br>1542.4	Hydro/Solvo thermal	85	3h	Methanol/ethane	Powder state washing<br>2h<br>3h<br>4h	(54)

Supplementary Table 2. A literature summary of some of the BET surface areas of HKUST-1 and the corresponding synthetic technique and conditions.

Supplementary Note 10. Cu-HKUST-1 samples

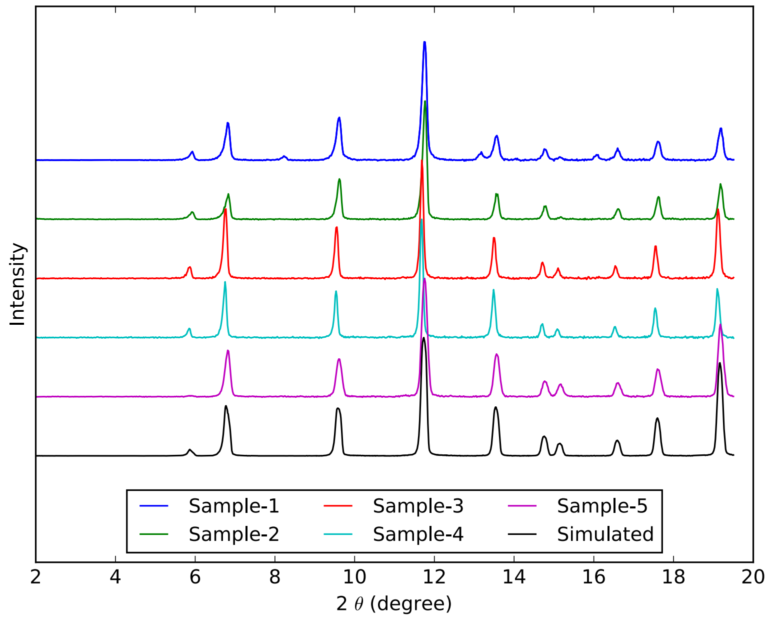

Supplementary Figure 9. Powder X-ray diffraction pattern of the five samples of Cu-HKUST-1with high crystallinity and wide range of BET surface area discussed in the manuscript.

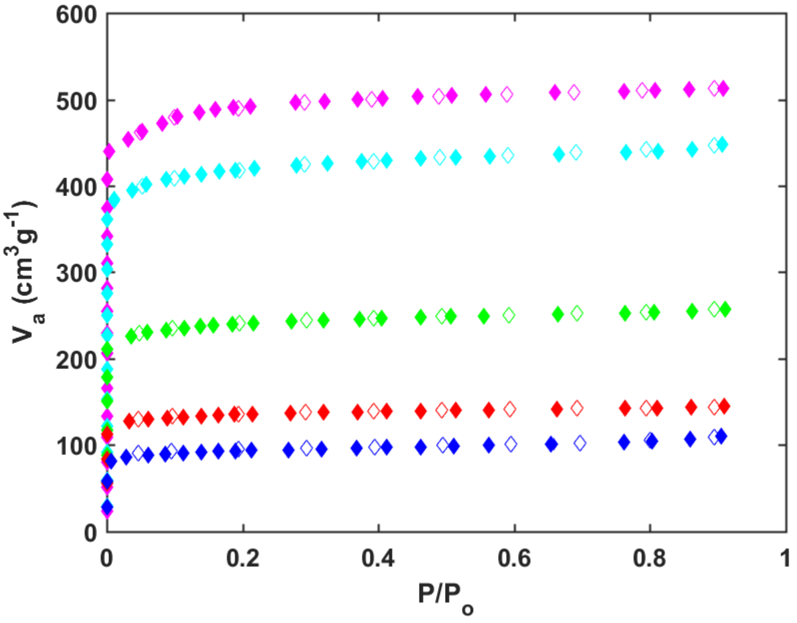

Supplementary Figure 10. The nitrogen adsorption isotherm at 77K for the five samples with high crystallinity (Cu-HKUST-1).

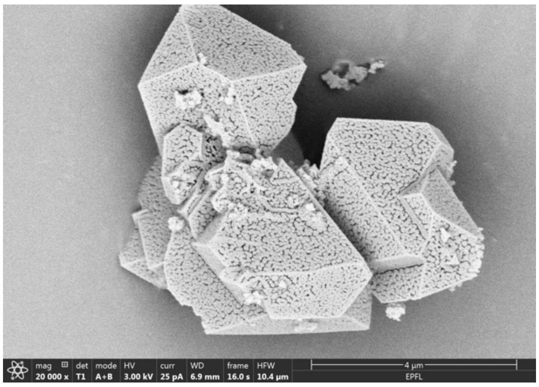

(a)

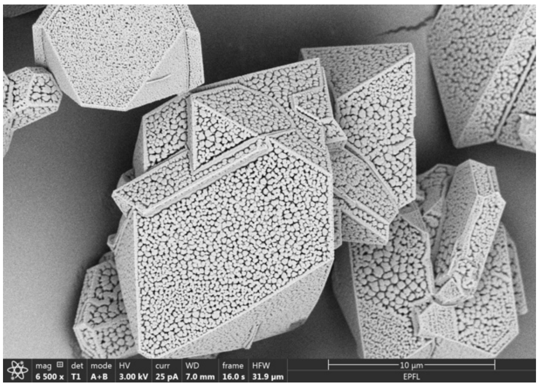

(b)

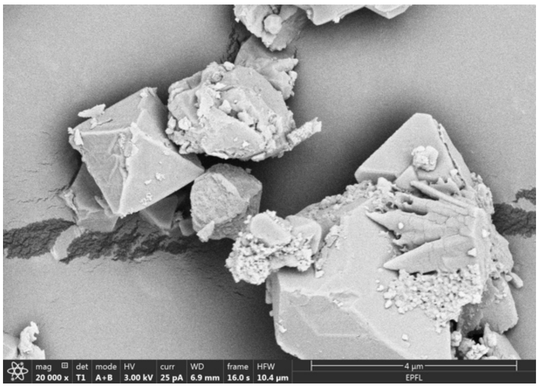

(c)

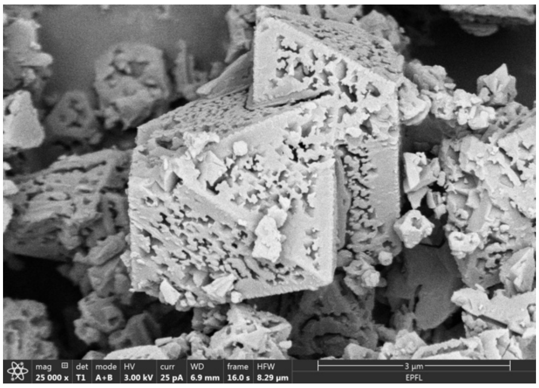

(d)

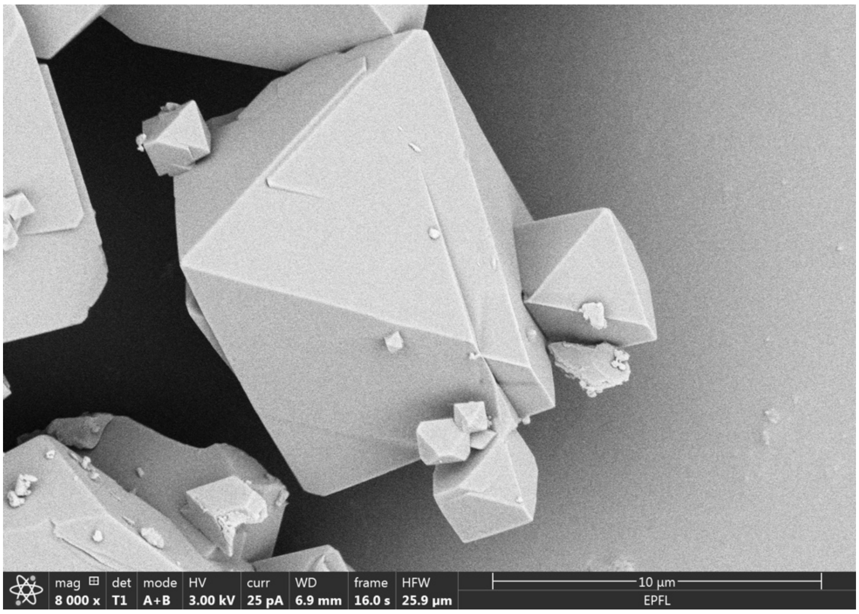

(e)

Supplementary Figure 11. More SEM images of the five samples with high crystallinity based on powder X-ray diffraction but wide range of BET (300-2121 $m^2 g^{-1}$*). (a), (b), (c), (d) and (e) are corresponding to sample 1 to 5, respectively.*

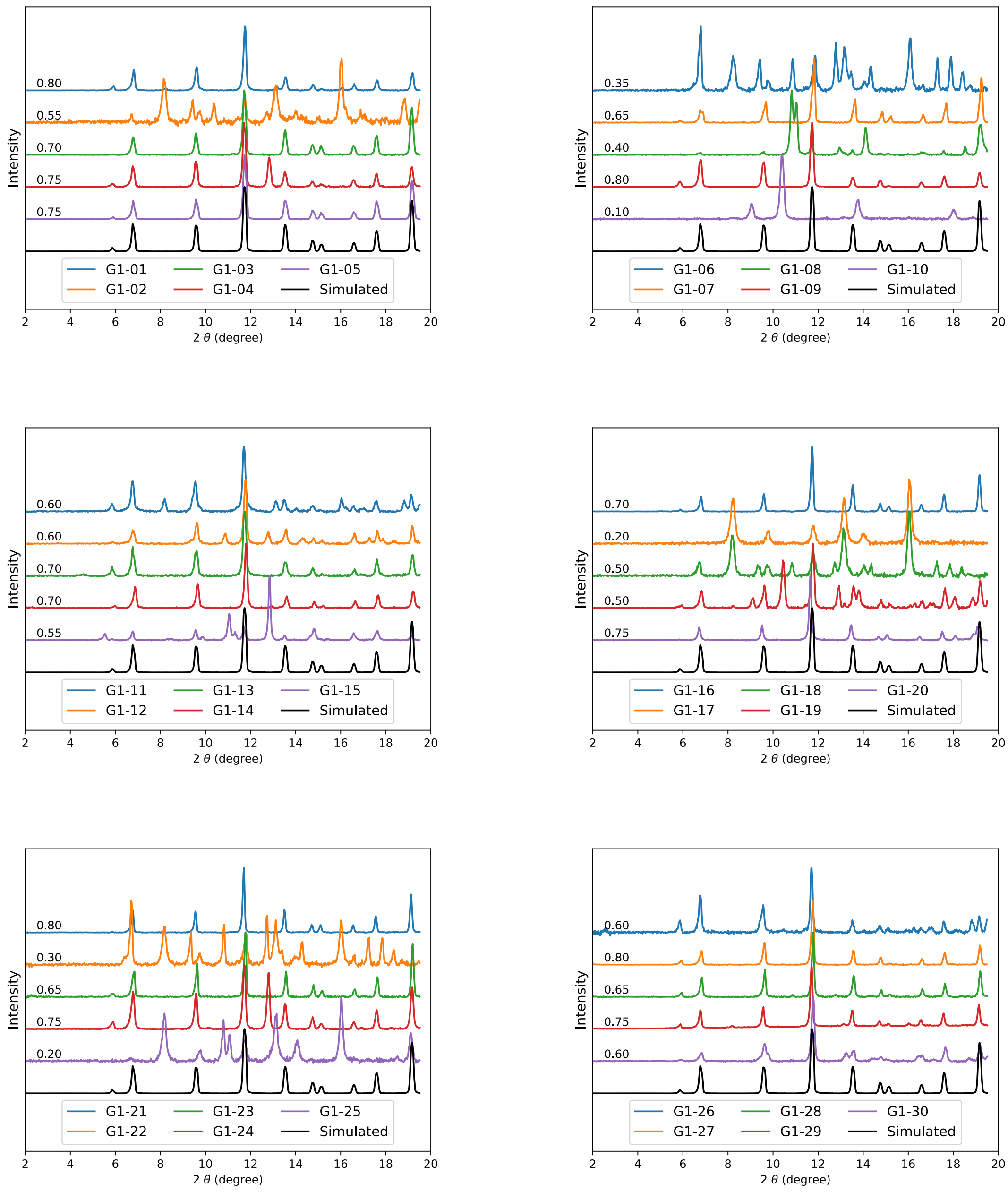

Supplementary Figure 12. The PXRDs of all samples in the first generation (G-1) of genetic algorithm optimisation of Cu-HKUST-1.

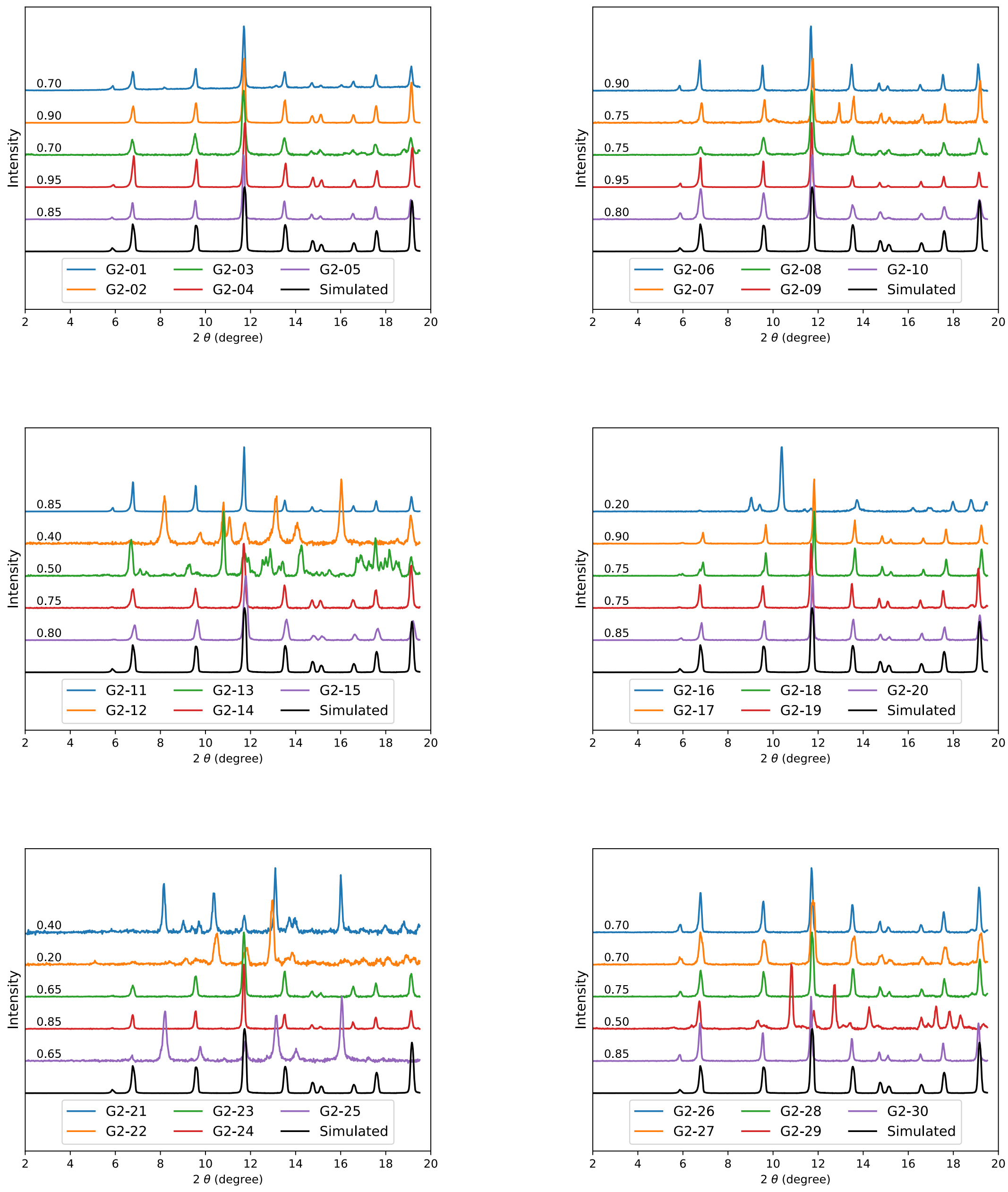

Supplementary Figure 13. The PXRDs of all samples in the second generation (G-2) of genetic algorithm optimisation of Cu-HKUST-1.

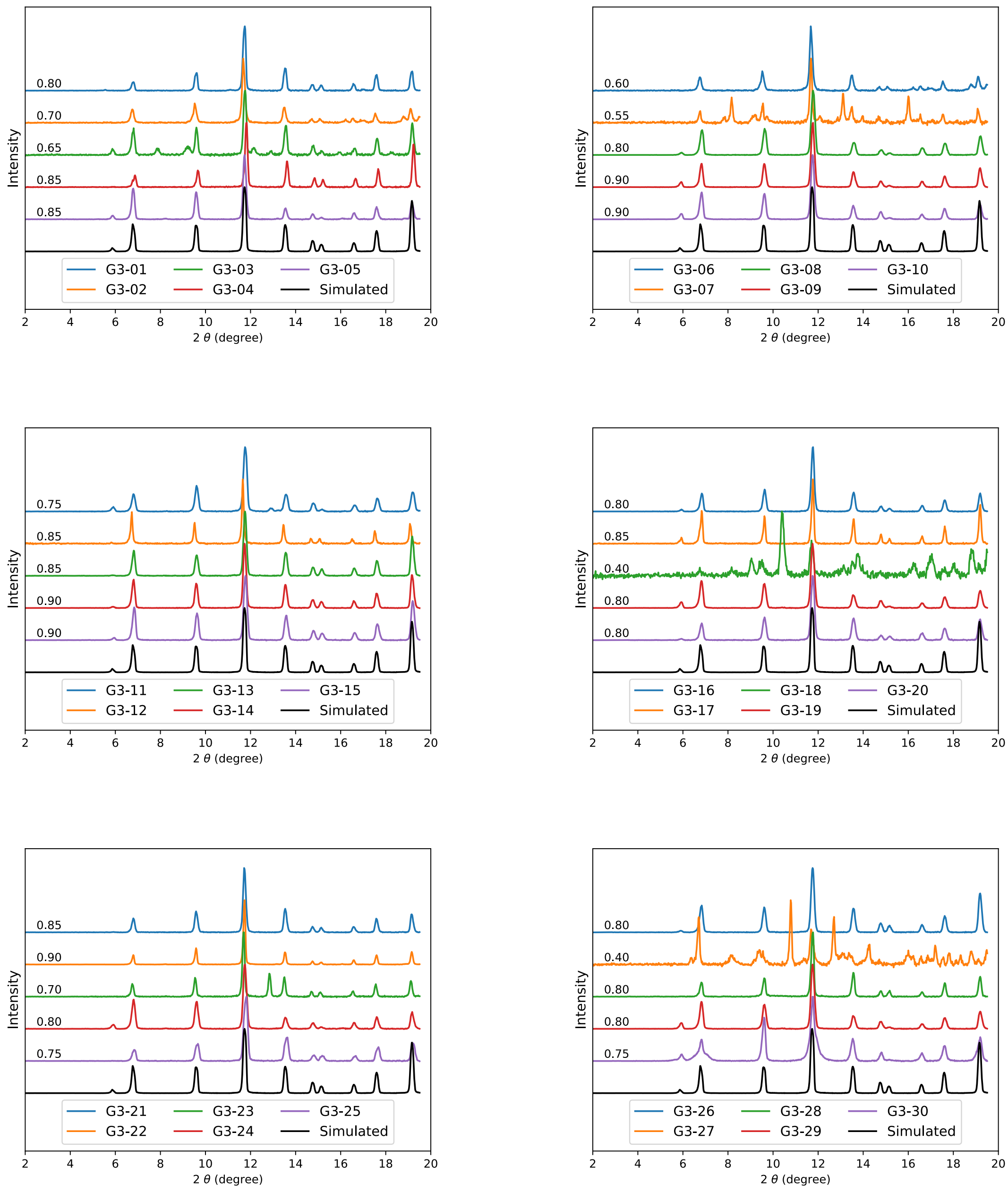

Supplementary Figure 14. The PXRDs of all samples in the third generation (G-3) of genetic algorithm optimisation of Cu-HKUST-1.

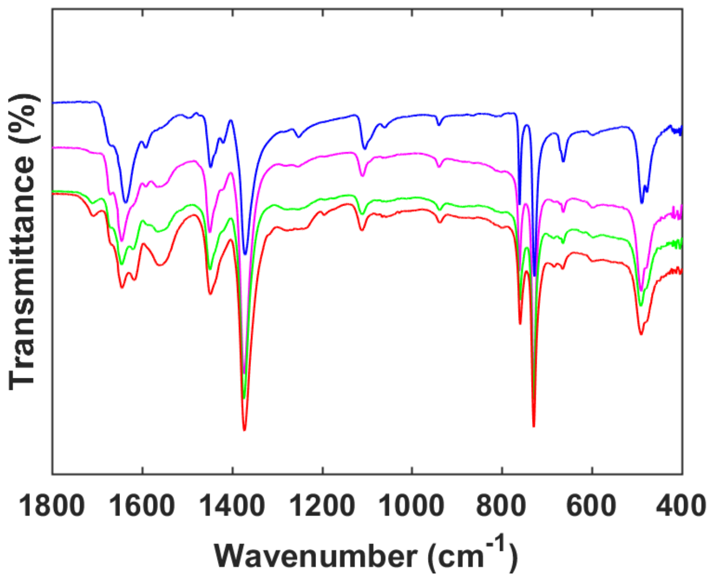

Supplementary Figure 15. FTIR spectra of the highly crystalline Cu-HKUST-1 MOFs.

FTIR spectra: The band at 1646 cm^{-1} which is observed in all the HKUST-1 MOFs can be assigned to the asymmetric stretching vibration due to the coordination of the carboxylate group of the BTC ligand and the bands observed at 1448 and 1372 cm^{-1} can be assigned to the symmetric stretching vibrations. The distinct band observed at 728 cm^{-1} relates to the Cu-O bond (metal-ligand coordination).

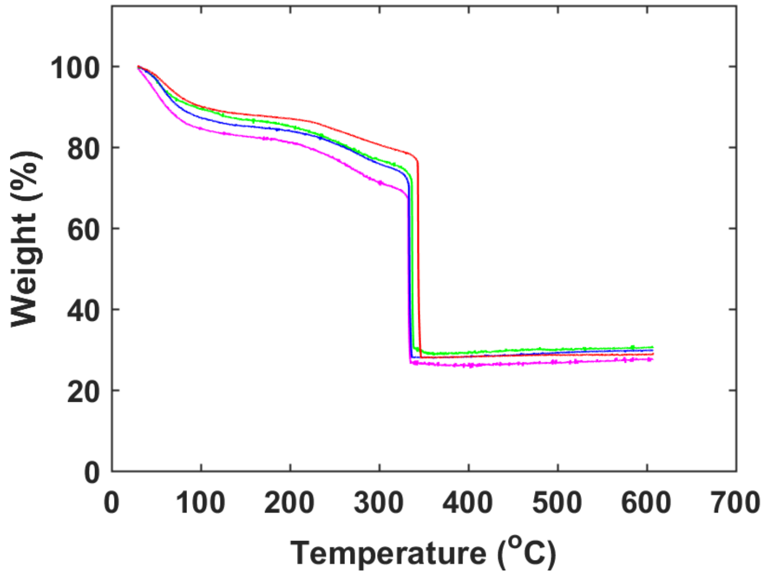

Supplementary Figure 16. Thermogravimetric analysis plots of the highly crystalline HKUST-1 MOFs.

Thermogravimetric analysis: the TGA curves exhibit several steps of weight losses. The initial two weight losses from 30 − 90°C and from 90 − 270°C can be correlated to the removal of moisture and solvent molecules. Further which, a sharp decrease in weight is observed which is attributed to the decomposition of the ligand. On further increase in temperature, there is no weight change (in air) with respect to the temperature.

	Chemical formula	Element	Calculated	Found
Sample 1	$[Cu_3C_{18}O_{15}H_{12}]\cdot(EtOH)\cdot(iPOH)\cdot3(H_2O)$	H	3.93	4.01
		C	33.72	32.99
Sample 3	$[Cu_3C_{18}O_{15}H_{12}]\cdot(DMF)\cdot(iPOH)\cdot3(H_2O)$	H	3.93	3.17
		C	34.06	33.84
		N	1.65	1.78
Sample 4	$[Cu_3C_{18}O_{15}H_{12}]\cdot2(DMF)\cdot2(H_2O)$	H	3.59	3.34
		C	34.27	34.07
		N	3.33	3.29
Sample 3	$[Cu_3C_{18}O_{15}H_{12}]\cdot3(DMF)\cdot2(H_2O)$	H	3.93	3.86
		C	36.18	35.86
		N	4.68	4.44

Supplementary Table 3. Elemental analysis of the HKUST-1 MOFs.

Supplementary Note 11. Zn-HKUST-1 samples

We performed experiments to synthesize Zn-HKUST-1 in three sets, successful conditions of Cu-HKUST-1, diverse set without chemical intuition and diverse set based on chemical intuition. Only the latter set yields the Zn-HKUST-1 powder. The synthesis conditions and the powder x-ray diffraction patterns of the three hits in the diverse set with chemical intuition are listed and shown in Supplementary Table 4 and *Supplementary Figure 17*, respectively. One needs to perform genetic algorithm optimisation to obtain phase pure and crystalline Zn-HKUST-1 by crossover and mutation of genes of the hits. Noteworthy to mention that it is impossible to start optimisation for the other sets, i.e. successful conditions of Cu-HKUST-1 and diverse set without chemical intuition, as there is no successful condition.

Sample	H_2O	DMF	EtOH	MeOH	iPrOH	Reactants Ratio	Temperature	Microwave power	Reaction time
1	0.0	5.5	0.0	0.0	0.5	1	102	155	29
2	3.0	0.0	2.5	0.0	0.0	1.5	171	150	5
3	0.0	2.5	0.0	0.0	3.0	1	100	250	19

Supplementary Table 4. The synthesis condition of the three hits for Zn-HKUST-1

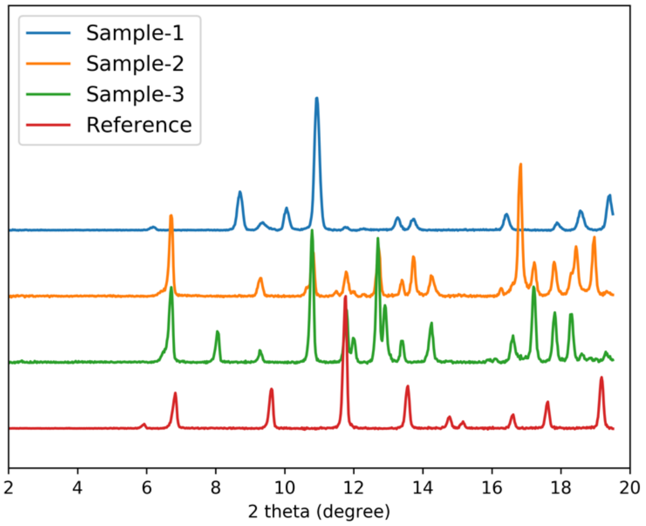

Supplementary Figure 17. Powder X-ray diffraction pattern for several hits in the diverse set based on importance of variables. Further optimization is required to get phase pure and crystalline materials.

Supplementary Note 12. Accuracy and reproducibility of experiments

The robotic platform provides a consistent synthesis protocol and a good control over the synthesis variables and reaction conditions. We automated the synthesis with programming the synthesis steps in the Chemspeed AutoSuite software (See *Supplementary Figure 18* for the details). As it can be seen in *Supplementary Figure 19* the amount of solvents and the solids (metal salt and the ligand) dispensed (refer to the numbers under the columns "actual volume", "actual quantity") on the robotic platform nearly match with the programmed amounts (refer to the numbers under the columns "entered volume", "entered quantity") for the solid dispensers and perfectly match for the liquid dispensers. This reveal the high accuracy and control over the synthesis variables and conditions.

To further investigate the reproducibility of the reaction outcomes, we performed synthesis of Cu-HKUST-1 for two of the discussed samples in the main manuscript and measured their BET surface area and collected their PXRDs. Supplementary Table 5 and *Supplementary Figure 20* summarize the satisfactory reproducibility of the outcome of these reactions.

Sample	BET [m^2g^{-1}]				Average
	Measurement 1	Measurement 2	Measurement 3	Measurement 4	
4	1585	1646	1549	1607	1596
5	2011	1947	2101	2121	2045

Supplementary Table 5. The measured BET surface area of the repeated experiments of two of the samples discussed in the manuscript.

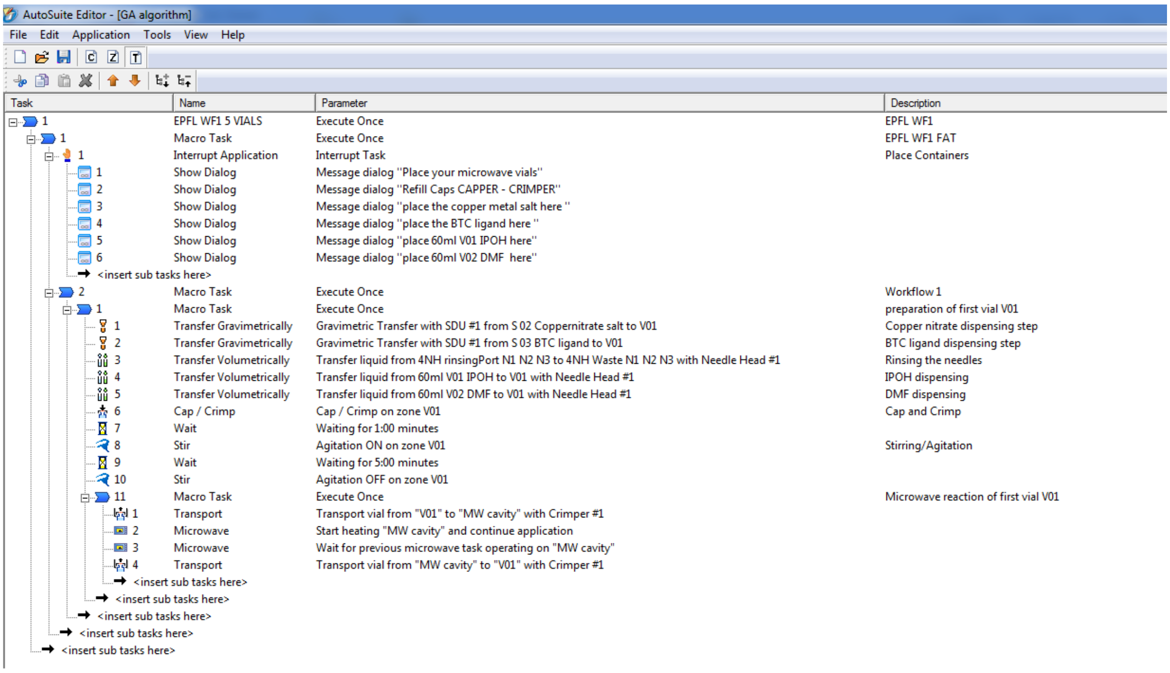

Supplementary Figure 18. A screenshot of the algorithm that was programmed with the Chemspeed AutoSuite software for the automated robotic synthesis of a specific HKUST-1 MOF showing the synthesis steps.

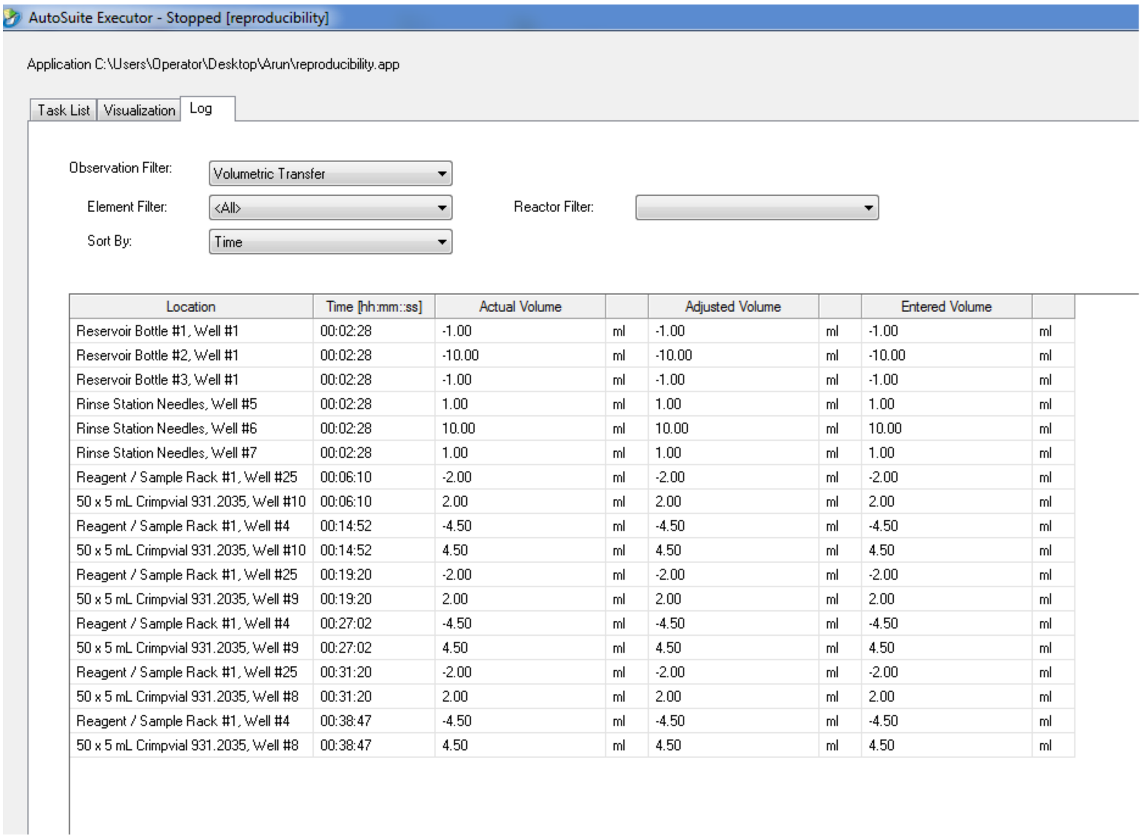

Location	Time [hh:mm::ss]	Actual Volume		Adjusted Volume		Entered Volume	
Reservoir Bottle #1, Well #1	00:02:28	-1.00	ml	-1.00	ml	-1.00	ml
Reservoir Bottle #2, Well #1	00:02:28	-10.00	ml	-10.00	ml	-10.00	ml
Reservoir Bottle #3, Well #1	00:02:28	-1.00	ml	-1.00	ml	-1.00	ml
Rinse Station Needles, Well #5	00:02:28	1.00	ml	1.00	ml	1.00	ml
Rinse Station Needles, Well #6	00:02:28	10.00	ml	10.00	ml	10.00	ml
Rinse Station Needles, Well #7	00:02:28	1.00	ml	1.00	ml	1.00	ml
Reagent / Sample Rack #1, Well #25	00:06:10	-2.00	ml	-2.00	ml	-2.00	ml
50 x 5 mL Crimpvial 931.2035, Well #10	00:06:10	2.00	ml	2.00	ml	2.00	ml
Reagent / Sample Rack #1, Well #4	00:14:52	-4.50	ml	-4.50	ml	-4.50	ml
50 x 5 mL Crimpvial 931.2035, Well #10	00:14:52	4.50	ml	4.50	ml	4.50	ml
Reagent / Sample Rack #1, Well #25	00:19:20	-2.00	ml	-2.00	ml	-2.00	ml
50 x 5 mL Crimpvial 931.2035, Well #9	00:19:20	2.00	ml	2.00	ml	2.00	ml
Reagent / Sample Rack #1, Well #4	00:27:02	-4.50	ml	-4.50	ml	-4.50	ml
50 x 5 mL Crimpvial 931.2035, Well #9	00:27:02	4.50	ml	4.50	ml	4.50	ml
Reagent / Sample Rack #1, Well #25	00:31:20	-2.00	ml	-2.00	ml	-2.00	ml
50 x 5 mL Crimpvial 931.2035, Well #8	00:31:20	2.00	ml	2.00	ml	2.00	ml
Reagent / Sample Rack #1, Well #4	00:38:47	-4.50	ml	-4.50	ml	-4.50	ml
50 x 5 mL Crimpvial 931.2035, Well #8	00:38:47	4.50	ml	4.50	ml	4.50	ml

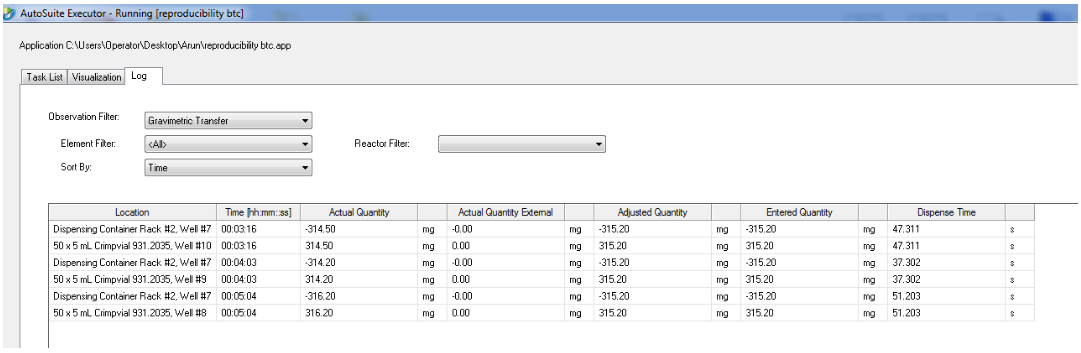

Location	Time [hh:mm::ss]	Actual Quantity		Actual Quantity External		Adjusted Quantity		Entered Quantity		Dispense Time	
Dispensing Container Rack #2, Well #7	00:03:16	-314.50	mg	-0.00	mg	-315.20	mg	-315.20	mg	47.311	s
50 x 5 mL Crimpvial 931.2035, Well #10	00:03:16	314.50	mg	0.00	mg	315.20	mg	315.20	mg	47.311	s
Dispensing Container Rack #2, Well #7	00:04:03	-314.20	mg	-0.00	mg	-315.20	mg	-315.20	mg	37.302	s
50 x 5 mL Crimpvial 931.2035, Well #9	00:04:03	314.20	mg	0.00	mg	315.20	mg	315.20	mg	37.302	s
Dispensing Container Rack #2, Well #7	00:05:04	-316.20	mg	-0.00	mg	-315.20	mg	-315.20	mg	51.203	s
50 x 5 mL Crimpvial 931.2035, Well #8	00:05:04	316.20	mg	0.00	mg	315.20	mg	315.20	mg	51.203	s

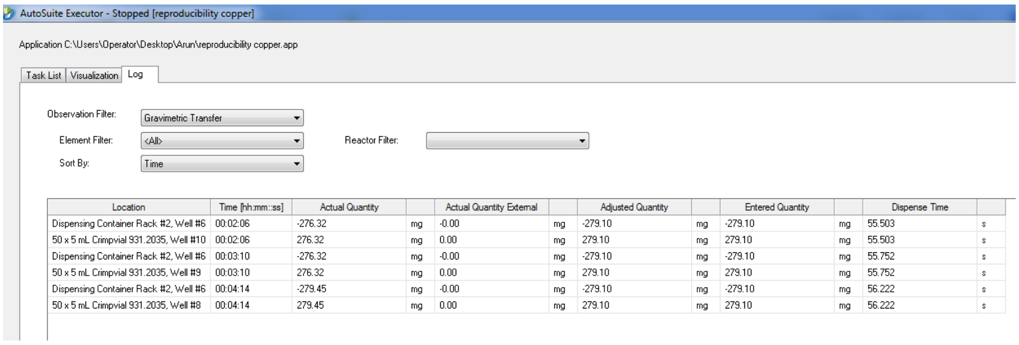

Location	Time [hh:mm::ss]	Actual Quantity		Actual Quantity External		Adjusted Quantity		Entered Quantity		Dispense Time	
Dispensing Container Rack #2, Well #6	00:02:06	-276.32	mg	-0.00	mg	-279.10	mg	-279.10	mg	55.503	s
50 x 5 mL Crimpvial 931.2035, Well #10	00:02:06	276.32	mg	0.00	mg	279.10	mg	279.10	mg	55.503	s
Dispensing Container Rack #2, Well #6	00:03:10	-276.32	mg	-0.00	mg	-279.10	mg	-279.10	mg	55.752	s
50 x 5 mL Crimpvial 931.2035, Well #9	00:03:10	276.32	mg	0.00	mg	279.10	mg	279.10	mg	55.752	s
Dispensing Container Rack #2, Well #6	00:04:14	-279.45	mg	-0.00	mg	-279.10	mg	-279.10	mg	56.222	s
50 x 5 mL Crimpvial 931.2035, Well #8	00:04:14	279.45	mg	0.00	mg	279.10	mg	279.10	mg	56.222	s

Supplementary Figure 19. Screenshots of the log files of the solvent dispenser and the solid dispenser for one HKUST-1 MOF synthetic reaction performed thrice. Well #10, #9, #8 refer to the microwave reaction vials placed in the aforementioned wells.

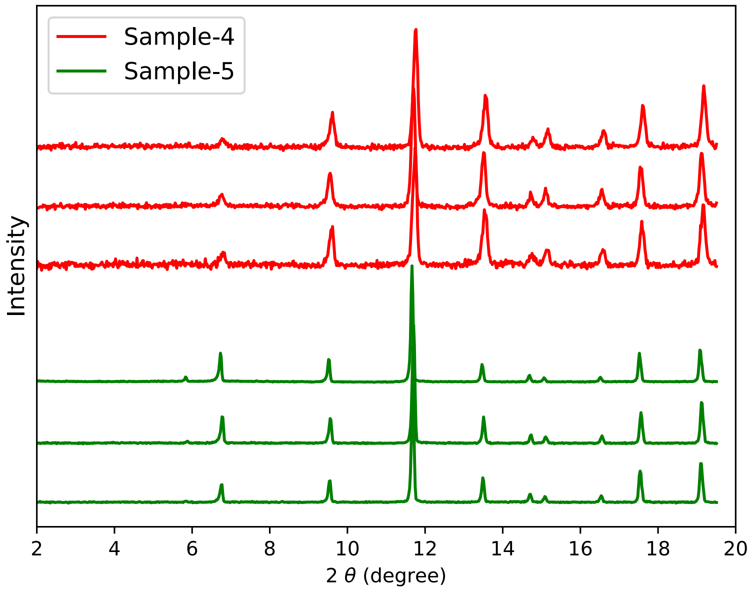

Supplementary Figure 20. The powder X-ray diffraction of the sample 4 and 5, repeated three times to evaluate the reproducibility of the synthesis and measurements.

Supplementary References

1. Davis L (ed. . Handbook of genetic algorithms. Van Nostrand Reinhold, New York. 1991;
2. MATLAB 2018a, Global Optimisation Toolbox and statistics and machine learning Toolbox. The MathWorks, Inc., Natick, Massachusetts, United States; 2018.
3. Cockcroft JK, Birkbeck College, London UK. Powder Diffraction Course Material [Internet]. Birkbeck College, London, United Kingdom. 1988. Available from: http://pd.chem.ucl.ac.uk
4. Jian Z, Hejing W. The physical meanings of 5 basic parameters for an X-ray diffraction peak and their application. Chinese J Geochemistry. 2003 Jan;22(1):38–44.
5. Dorset DL. X-ray Diffraction: A Practical Approach. Microsc Microanal. 1998 Oct 28;4(05):513–5.
6. Polinsky A, Feinstein RD, Shi S, Kuki A. LiBrain: software for automated design of exploratory and targeted combinatorial libraries. Mol Divers Comb Chem Libr Drug Discov. 1996;996:219–32.
7. Alpaydin E. Introduction to machine learning. MIT press; 2014.
8. Liaw Merck A, Liaw A, Wiener M. Classification and Regression by randomForest. 2002;23.
9. Moosavi, Seyed Mohamad, Chidambaram, Arunraj, Talirz, Leopold, Haranczyk, Maciej, Stylianou, Kyriakos C., Smit B. Capturing chemical intuition in synthesis of metal-organic frameworks [Internet]. Materials Cloud Archive. 2018. Available from: doi: 10.24435/materialscloud:2018.0011/v1
10. Fortin F-A, Rainville F-M De, Gardner M-A, Parizeau M, Gagné C. DEAP: Evolutionary Algorithms Made Easy. J Mach Learn Res. 2012;13(Jul):2171–5.
11. Pedregosa F, Varoquaux G, Gramfort A, Michel V, Thirion B, Grisel O, et al. Scikit-learn: Machine Learning in Python. J Mach Learn Res. 2011;12(Oct):2825–30.

12. Guyon I, Elisseeff A. An Introduction to Variable and Feature Selection. J Mach Learn Res. 2003;3(3):1157–82.

13. Saeys Y, Inza I, Larrañaga P. A review of feature selection techniques in bioinformatics. Bioinformatics. 2007;23(19):2507–17.

14. Multidimensional Scaling - Joseph B. Kruskal, Myron Wish.

15. Modern Multidimensional Scaling: Theory and Applications - I. Borg, P. J. F. Groenen.

16. Ceriotti M, Tribello GA, Parrinello M. Simplifying the representation of complex free-energy landscapes using sketch-map. Proc Natl Acad Sci U S A. 2011 Aug 9;108(32):13023–8.

17. Chui SS-Y, Lo SM-F, Charmant JPH, Orpen AG, Williams ID. A chemically functionalizable nanoporous material [Cu3 (TMA) 2 (H2O) 3] n. Science (80-). 1999;283(5405):1148–50.

18. Biemmi E, Christian S, Stock N, Bein T. High-throughput screening of synthesis parameters in the formation of the metal-organic frameworks MOF-5 and HKUST-1. Microporous Mesoporous Mater. 2009;117(1–2):111–7.

19. Chen L, Reiss PS, Chong SY, Holden D, Jelfs KE, Hasell T, et al. Separation of rare gases and chiral molecules by selective binding in porous organic cages. Nat Mater. 2014;13(10):954–60.

20. Moellmer J, Moeller A, Dreisbach F, Glaeser R, Staudt R. High pressure adsorption of hydrogen, nitrogen, carbon dioxide and methane on the metal–organic framework HKUST-1. Microporous Mesoporous Mater. 2011 Feb 1;138(1–3):140–8.

21. Krawiec P, Kramer M, Sabo M, Kunschke R, Fröde H, Kaskel S. Improved Hydrogen Storage in the Metal-Organic Framework Cu3(BTC)2. Adv Eng Mater. 2006 Apr;8(4):293–6.

22. Klein N, Henschel A, Kaskel S. n-Butane adsorption on Cu3(btc)2 and MIL-101.

Microporous Mesoporous Mater. 2010 Apr 1;129(1–2):238–42.

23. Wee LH, Lohe MR, Janssens N, Kaskel S, Martens JA. Fine tuning of the metal–organic framework Cu3(BTC)2 HKUST-1 crystal size in the 100 nm to 5 micron range. J Mater Chem. 2012 Jun 19;22(27):13742.

24. Lin K-S, Adhikari AK, Ku C-N, Chiang C-L, Kuo H. Synthesis and characterization of porous HKUST-1 metal organic frameworks for hydrogen storage. Int J Hydrogen Energy. 2012 Sep 1;37(18):13865–71.

25. Chowdhury P, Mekala S, Dreisbach F, Gumma S. Adsorption of CO, CO2 and CH4 on Cu-BTC and MIL-101 metal organic frameworks: Effect of open metal sites and adsorbate polarity. Microporous Mesoporous Mater. 2012 Apr 1;152:246–52.

26. Jinchen Liu †, Jeffrey T. Culp ‡, Sittichai Natesakhawat §, Bradley C. Bockrath §, Brian Zande ⊥, S. G. Sankar ⊥, et al. Experimental and Theoretical Studies of Gas Adsorption in Cu3(BTC)2: An Effective Activation Procedure. 2007;

27. Rowsell JLC, Yaghi OM. Effects of Functionalization, Catenation, and Variation of the Metal Oxide and Organic Linking Units on the Low-Pressure Hydrogen Adsorption Properties of Metal−Organic Frameworks. J Am Chem Soc. 2006 Feb 1;128(4):1304–15.

28. Wiersum AD, Chang J-S, Serre C, Llewellyn PL. An Adsorbent Performance Indicator as a First Step Evaluation of Novel Sorbents for Gas Separations: Application to Metal–Organic Frameworks. Langmuir. 2013 Mar 12;29(10):3301–9.

29. Senkovska I, Kaskel S. High pressure methane adsorption in the metal-organic frameworks Cu3(btc)2, Zn2(bdc)2dabco, and Cr3F(H2O)2O(bdc)3. Microporous Mesoporous Mater. 2008 Jul 1;112(1–3):108–15.

30. Küsgens P, Rose M, Senkovska I, Fröde H, Henschel A, Siegle S, et al. Characterization of metal-organic frameworks by water adsorption. Microporous Mesoporous Mater. 2009 Apr 15;120(3):325–30.

31. Münch AS, Mertens FORL. HKUST-1 as an open metal site gas chromatographic stationary phase—capillary preparation, separation of small hydrocarbons and electron donating compounds, determination of thermodynamic data. J Mater Chem. 2012 May 1;22(20):10228.
32. Zhao Z, Wang S, Yang Y, Li X, Li J, Li Z. Competitive adsorption and selectivity of benzene and water vapor on the microporous metal organic frameworks (HKUST-1). Chem Eng J. 2015 Jan 1;259:79–89.
33. Huo J, Brightwell M, El Hankari S, Garai A, Bradshaw D. A versatile, industrially relevant, aqueous room temperature synthesis of HKUST-1 with high space-time yield. J Mater Chem A. 2013 Nov 19;1(48):15220.
34. Ye S, Jiang X, Ruan L-W, Liu B, Wang Y-M, Zhu J-F, et al. Post-combustion CO2 capture with the HKUST-1 and MIL-101(Cr) metal–organic frameworks: Adsorption, separation and regeneration investigations. Microporous Mesoporous Mater. 2013 Sep 15;179:191–7.
35. Chen H, Wang L, Yang J, Yang RT. Investigation on Hydrogenation of Metal–Organic Frameworks HKUST-1, MIL-53, and ZIF-8 by Hydrogen Spillover. J Phys Chem C. 2013 Apr 18;117(15):7565–76.
36. Wong-Foy AG, Matzger AJ, Yaghi OM. Exceptional H2 Saturation Uptake in Microporous Metal−Organic Frameworks. J Am Chem Soc. 2006 Mar 1;128(11):3494–5.
37. Yan X, Komarneni S, Zhang Z, Yan Z. Extremely enhanced CO2 uptake by HKUST-1 metal–organic framework via a simple chemical treatment. Microporous Mesoporous Mater. 2014 Jan 1;183:69–73.
38. Rocío-Bautista P, Martínez-Benito C, Pino V, Pasán J, Ayala JH, Ruiz-Pérez C, et al. The metal–organic framework HKUST-1 as efficient sorbent in a vortex-assisted dispersive micro solid-phase extraction of parabens from environmental waters,

cosmetic creams, and human urine. Talanta. 2015 Jul 1;139:13–20.

39. Raganati F, Gargiulo V, Ammendola P, Alfe M, Chirone R. CO2 capture performance of HKUST-1 in a sound assisted fluidized bed. Chem Eng J. 2014 Mar 1;239:75–86.

40. Al-Janabi N, Hill P, Torrente-Murciano L, Garforth A, Gorgojo P, Siperstein F, et al. Mapping the Cu-BTC metal–organic framework (HKUST-1) stability envelope in the presence of water vapour for CO2 adsorption from flue gases. Chem Eng J. 2015 Dec 1;281:669–77.

41. Min Wang Q, Shen D, Bülow M, Ling Lau M, Deng S, Fitch FR, et al. Metallo-organic molecular sieve for gas separation and purification. Microporous Mesoporous Mater. 2002 Sep 16;55(2):217–30.

42. Millward AR, Yaghi OM. Metal−Organic Frameworks with Exceptionally High Capacity for Storage of Carbon Dioxide at Room Temperature. J Am Chem Soc. 2005 Dec 1;127(51):17998–9.

43. Panella B, Hirscher M, Pütter H, Müller U. Hydrogen Adsorption in Metal–Organic Frameworks: Cu-MOFs and Zn-MOFs Compared. Adv Funct Mater. 2006 Mar 3;16(4):520–4.

44. Hartmann M, Kunz S, Himsl D, Tangermann O, Ernst S, Wagener A. Adsorptive Separation of Isobutene and Isobutane on $Cu_3(BTC)_2$. Langmuir. 2008 Aug;24(16):8634–42.

45. Schlesinger M, Schulze S, Hietschold M, Mehring M. Evaluation of synthetic methods for microporous metal–organic frameworks exemplified by the competitive formation of [Cu2(btc)3(H2O)3] and [Cu2(btc)(OH)(H2O)]. Microporous Mesoporous Mater. 2010 Jul 1;132(1–2):121–7.

46. Seo Y-K, Hundal G, Jang IT, Hwang YK, Jun C-H, Chang J-S. Microwave synthesis of hybrid inorganic–organic materials including porous Cu3(BTC)2 from Cu(II)-trimesate mixture. Microporous Mesoporous Mater. 2009 Mar 1;119(1–3):331–7.

47. Li Y, Yang RT. Hydrogen storage in metal-organic and covalent-organic frameworks by spillover. AIChE J. 2008 Jan;54(1):269–79.

48. Li Z-Q, Qiu L-G, Xu T, Wu Y, Wang W, Wu Z-Y, et al. Ultrasonic synthesis of the microporous metal–organic framework Cu3(BTC)2 at ambient temperature and pressure: An efficient and environmentally friendly method. Mater Lett. 2009 Jan 15;63(1):78–80.

49. Bhunia MK, Hughes JT, Fettinger JC, Navrotsky A. Thermochemistry of Paddle Wheel MOFs: Cu-HKUST-1 and Zn-HKUST-1. Langmuir. 2013 Jun 25;29(25):8140–5.

50. Kim HK, Yun WS, Kim M-B, Kim JY, Bae Y-S, Lee J, et al. A Chemical Route to Activation of Open Metal Sites in the Copper-Based Metal–Organic Framework Materials HKUST-1 and Cu-MOF-2. J Am Chem Soc. 2015 Aug 12;137(31):10009–15.

51. Wang F, Guo H, Chai Y, Li Y, Liu C. The controlled regulation of morphology and size of HKUST-1 by "coordination modulation method." Microporous Mesoporous Mater. 2013 Jun 1;173:181–8.

52. Elsaidi SK, Ongari D, Xu W, Mohamed MH, Haranczyk M, Thallapally PK. Xenon Recovery at Room Temperature using Metal-Organic Frameworks. Chem - A Eur J. 2017 Aug 10;23(45):10758–62.

53. Chowdhury P, Bikkina C, Meister D, Dreisbach F, Gumma S. Comparison of adsorption isotherms on Cu-BTC metal organic frameworks synthesized from different routes. Microporous Mesoporous Mater. 2009 Jan 1;117(1–2):406–13.

54. Mu X, Chen Y, Lester E, Wu T. Optimized synthesis of nano-scale high quality HKUST-1 under mild conditions and its application in CO2 capture. Microporous Mesoporous Mater. 2018 Nov 1;270:249–57.
