# Supplementary material for: Capturing chemical intuition in synthesis of metal-organic frameworks
Source: Nat Commun. 2019 Feb 1;10:539. doi: 10.1038/s41467-019-08483-9 (PMC6358622; doi:10.1038/s41467-019-08483-9)
Supplement: Supplementary file 3 — Description of Additional Supplementary Files [file 41467_2019_8483_MOESM3_ESM.pdf]

Description of Additional Supplementary Files

File Name: Supplementary Movie 1
Description: Sy-Co-Finder web application. This video shows the web application developed in this study.

File Name: Supplementary Data 1
Description: The synthesis conditions and their corresponding fitness scores of the synthesis trials of the three generations of the genetic algorithm optimization for Cu-HKUST1.
